# Identification of the *Plasmodium* species in clinical samples from children residing in five epidemiological strata of malaria in Cameroon

**DOI:** 10.1186/s41182-017-0058-5

**Published:** 2017-06-15

**Authors:** Tebit Emmanuel Kwenti, Tayong Dizzle Bita Kwenti, Longdoh Anna Njunda, Andreas Latz, Kukwah Anthony Tufon, Theresa Nkuo-Akenji

**Affiliations:** 1 Department of Medical Laboratory Sciences, University of Buea, P.B. 63, Buea, Cameroon; 2 Department of Microbiology and Parasitology, University of Buea, P.B. 63, Buea, Cameroon; 3 Diagnostic laboratory, Regional Hospital of Buea, P.B. 32, Buea, Cameroon; 4 Research and Development Department, NovaTec Immundiagnostica GmbH, Dietzenbach, Germany

**Keywords:** Plasmodium species, PCR, Microscopy, Malaria, Children, Epidemiological strata, Cameroon

## Abstract

**Background:**

Malaria in Cameroon was previously known to be caused solely by *Plasmodium falciparum* but today, evidence points to other *Plasmodium* species including *P. vivax*, *P. ovale* and *P. malariae*. The purpose of this study was to identify the *Plasmodium* species in clinical samples from children residing in five epidemiological strata of malaria in Cameroon, so as to advise control policies.

**Methods:**

One thousand six hundred nine febrile children (≤15 years) were recruited from five epidemiological strata of malaria including the Sudano-sahelian (SS) strata, the High inland plateau (HIP) strata, the South Cameroonian Equatorial forest (SCEF) strata, the High western plateau (HWP) strata and the Coastal (C) strata. Malaria parasites were detected by Giemsa microscopy (GM) while a multiplex polymerase chain reaction (PCR) was used to identify the *Plasmodium* species. Statistical analysis performed included the Pearson chi-square test, and statistical significance was set at *p* < 0.05.

**Results:**

The PCR-adjusted prevalence of malaria was 17.6%. The detection rate of PCR was higher than GM (*p* = 0.05). However, GM demonstrated a high sensitivity (85.5%) and specificity (100%) and, overall, a perfectly correlated agreement with PCR (97.5%). The prevalence of malaria was significantly higher in children between 60 and 119 months (*p* < 0.001) and in Limbe (in the Coastal strata) (*p* < 0.001). Contrariwise, the prevalence of malaria was not associated with gender (*p* = 0.239). *P. falciparum* was identified in all (100%) the cases of malaria; *P. ovale*, *P. vivax*, *P. malariae* and *P. knowlesi* were all absent. No case of mixed infection was identified.

**Conclusions:**

*P. falciparum* was the only species causing clinical malaria in the target population, which is contrary to studies that have reported *P. vivax*, *P. malariae* and *P. ovale* as causing clinical malaria in Cameroon.

## Background

Malaria remains a significant public health problem in many countries of the world especially in sub-Saharan Africa (SSA). The WHO estimates that in 2015, there were 214 million cases of malaria and 438,000 deaths attributed to malaria [1]. The majority (90%) of cases and death attributed to malaria occurs in SSA, especially in children aged under 5 years [2]. Although there has been a recent decline in the incidence of malaria, it is still a major killer in children, claiming the life of a child every 2 min [1] in SSA.

In Cameroon, malaria is the major cause of morbidity and mortality among the most vulnerable groups including children aged under 5 years (18%), pregnant women (5%), people living with HIV/AIDS (5.5%) and the poor (40%) [3, 4]. In Cameroon, malaria accounts for 48% of all hospital admissions, 30% of morbidity and 67% of childhood mortality per year [5, 6]. The epidemiology of malaria in Cameroon has been described as unique, having all the different epidemiological strata present in all of Africa [7, 8]. Six epidemiological strata have been identified and mapped in Cameroon, namely, the Sudano-sahelian (SS) strata, High inland plateau (HIP) strata, Savannah-forest transmission (SF) strata, South Cameroon Equatorial forest (SCEF) strata, High western plateau altitude (HWP) strata and Coastal (C) strata [7]. These epidemiologic strata differ in terms of their geographical and ecological characteristics, transmission pattern and endemicity level and in terms of the main vectors transmitting malaria parasites [7].

Malaria is caused by parasitic protozoans of the genus *Plasmodium*. Five species of Plasmodia are known to cause disease in humans, namely, *P. falciparum*, *P. ovale*, *P. malariae*, *P. vivax* and *P. knowlesi*. The distribution of the different Plasmodia is not uniform throughout the world; *P. vivax* is more predominant in Asia [9] and *P. falciparum* in Africa [10]. Although *P. falciparum* is the most virulent species accounting for the majority of deaths [11], recent evidence suggest that *P. vivax* malaria is also associated with potentially life-threatening conditions about as often as with a diagnosis of *P. falciparum* infection [12]. In Cameroon, malaria was known to be caused solely by *P. falciparum*, but an increasing number of studies performed recently report other species of Plasmodia including *P. vivax* which is the second major species occurring as either single or mixed infection with *P. falciparum* [13, 14]. *P. malariae* and *P. ovale* occur at a lower frequency and in most cases as mixed infection with *P. falciparum* [15–17]. *P. knowlesi* has not been reported to cause malaria in Cameroon. *P. knowlesi* which was only recently observed to cause human malaria [18] is the most common species today in certain areas of Southeast Asia [19]. Like *P. vivax*, *P. knowlesi* also requires the Duffy antigen for infection [20] and is therefore absent in populations lacking the Duffy antigen especially in SSA. Interestingly, *P. vivax* has been shown to infect some people lacking the Duffy antigen in Cameroon [13, 14]. With the increase in global travel, there is the possibility that *P. knowlesi* may as well evolve to infect Duffy-negative populations.

Studies to identify the *Plasmodium* species causing malaria in children in Cameroon are limited. With the increasing number of reports of malaria being caused by *Plasmodium* species other than falciparum in Cameroon, it is important to provide baseline data on the distribution of the different *Plasmodium* species associated with clinical malaria in children, which will inform control policies. This study was therefore designed to identify the *Plasmodium* species associated with clinical malaria in children residing in five epidemiological strata of malaria in Cameroon.

## Methods

### Study area

**Fig. 1:**
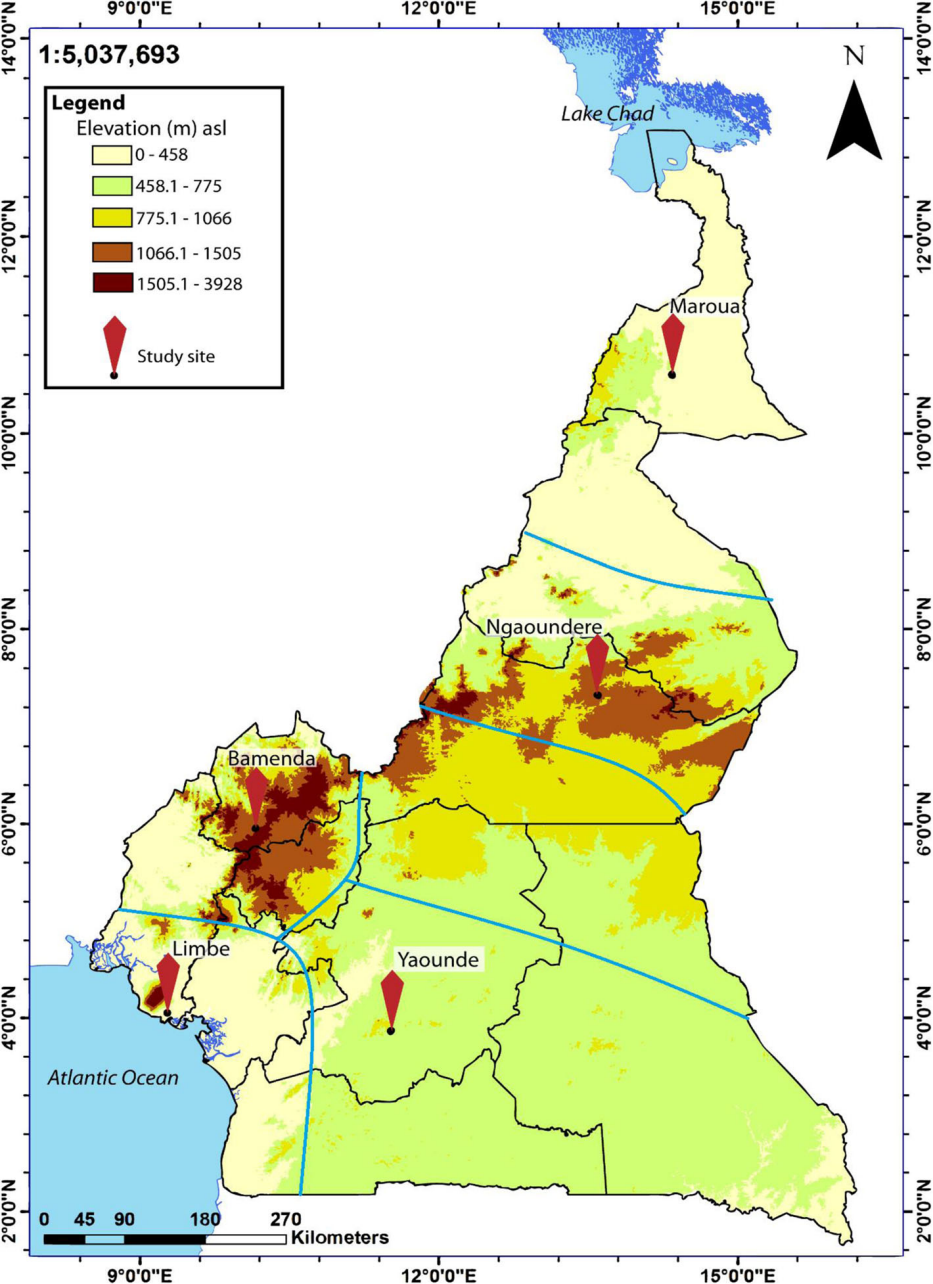
Map depicting the study sites selected. Five epidemiological strata are delineated

Five out of the six epidemiological strata of malaria in Cameroon were randomly selected for this study. Five study sites, each representing the epidemiological strata, were further selected and included: Maroua in the Sudano-sahelian (SS) strata, Ngaoundere in the High inland plateau (HIP) strata, Yaounde in the South Cameroonian Equatorial forest (SCEF) strata, Bamenda in the High western plateau (HWP) strata and Limbe in the Coastal (C) strata (Fig. 1). The characteristics of these epidemiological strata of malaria had previously been described [7].

### Study design and duration

This study was a hospital-based cross-sectional study involving children who came to consult in the outpatient department (OPD)/emergency units of regional hospitals in the different study sites. Data was collected between May and November 2015 (to coincide with the rainy season during which transmission is highest), simultaneously in the different study sites.

### Sample size estimation

The sample size was estimated using the following formula for sample size calculation as described by Swinscow [21];

$$ n = \frac{Z^2 x\  p\left(1- p\right)}{e^2} $$

$$ Z = 1.96 $$

*p* = prevalence of malaria in Cameroon which is 29% [22].

$$ e = \mathrm{error}\ \mathrm{rate} = 0.05 $$

$$ n = \frac{1.96^2 x\ 0.29\left(1-0.29\right)}{0.05^2}\kern0.75em  = 316.4\approx 317 $$

Thus, we recruited 317 participants per study site giving an overall total of 1585 for the 5 sites.

### Study population

Eligible participants were febrile children (≤15 years) who were consecutively recruited as they came to consult in the OPD or emergency unit of the hospitals in the different study sites. Patients not on any anti-malarial treatment for at least 1 week prior to the study commencing were included.

### Laboratory analyses

#### Specimen collection

About 4 ml of blood was collected from consented participants using aseptic techniques into EDTA test tubes. The uncoagulated blood was used to perform complete blood count (CBC) and prepare thick and thin films for malaria screening by microscopy and for the performance of polymerase chain reaction (PCR).

#### Performance of complete blood count

The CBC was performed using the Mindray® Auto haematology analyzer (BC-2800, Shenzhen Mindray Bio-Medical Electronics Co., Ltd). The white blood cell (WBC) counts were obtained from the CBC results and used in the estimation of malaria parasite density.

#### Microscopic detection of malaria parasite

The prepared blood films were air-dried and stained with 10% Giemsa (1 in 20 dilutions) for 25–30 min [23]. Blood films were read by two expert microscopists who were blinded from the results of the other. In case of any discrepancy with the results obtained by the two microscopists, a third was brought in and the results obtained were considered as final. Thick films were screened for at least 200 fields using a ×100 (oil immersion) objective. If asexual stages of malaria parasites were seen, they were counted until 500WBC were reached. The slides were only declared negative after counting to 2500WBC. Malaria parasite density was estimated by dividing the parasites counted by 500 and then multiplied by the WBC count of the participants to give numbers in parasite per microliter of blood [24].

### PCR detection of *Plasmodium* spp. in the blood

#### DNA extraction

The Chelex method [25] was used to extract DNA from the whole blood. Briefly, to 50 μl of whole blood, 0.5 ml of RBC lysis buffer (0.2% NaCl, 1% Triton X-100 and 1 mM EDTA) was added and mixed by inverting. The tubes and their contents were centrifuged at 11300 rpm for 10 min, and the supernatants were discarded. Five hundred microliters of 1× PBS were again added to wash the pellets and centrifuged again. The supernatants were discarded, and 50 μl of 20× Chelex and 150 μl of distilled water were added to the pellets. The tubes were then incubated at 99 °C for 20 min, centrifuged at maximum speed (13,500 rpm) for 10 min, and about 120 μl of the supernatant was collected and stored as parasite genomic DNA at −20 °C for future use.

#### Amplification of *Plasmodium* DNA

Genus- and species-specific sequences have been identified within the small subunit ribosomal DNA genes of all human malaria parasites (*P. falciparum*, *P. vivax*, *P. malariae*, *P. ovale and P. knowlesi*). We used the Oligonucleotide primer pairs designed by Snounou et al. [26] and Padley et al. [27] in a multiplex PCR to detect *P. falciparum*, *P. vivax*, *P. malariae* and *P. ovale* in the host’s total DNA. Reaction mix was made in a final volume of 12.5 μL containing 6.25 μL of 2× GoTaq®Hot Start Green master mix (Promega®, USA), 0.2 μM of each primer (IDT®, Iowa, USA), 3.25 μL of molecular grade water (SigmaAldrich, Switzerland) and 2.5 μL of DNA solution served as a template. Positive controls were included for all *Plasmodium* species. The cycling conditions were as follows: initial heating at 95 °C for 2 min, 43 cycles of heating at 95 °C for 45 s, annealing at 58 °C for 90 s and extension at 72 °C for 60 s, and a final extension at 72 °C for 5 min.

Amplification of *P. knowlesi* genomic DNA was done using the protocol described by Lucchi et al [28]. The reaction mix was made in a final volume of 25 μL containing 12.5 μL of 2× GoTaq®Hot Start Green master mix (Promega®, USA), 250 nM each of the forward and reverse primers (IDT®, Iowa, USA), molecular grade water (SigmaAldrich, Switzerland) and 1 μL of DNA as template. The cycling conditions were initial heating at 95 °C for 2 min, 35 cycles of heating at 95 °C for 30 s, annealing at 57 °C for 30 s and extension at 72 °C for 45 s, and a final extension at 72 °C for 5 min.

The PCR products were all analysed using 2% agarose gel electrophoresis, stained with ethidium bromide for fluorescence. The expected band sizes were 200 bp for *P. knowlesi*, 395 bp for *P. falciparum*, 499 bp for *P. vivax*, 269 bp for *P. malariae* and 436 bp for *P. ovale.* The samples were ran against a 100-bp molecular weight marker.

### Data analysis

Data collected were entered into an Excel spreadsheet and analysed using the Stata® version 12.1 software (StataCorp LP, Texas, USA). The statistical tests performed included the Pearson’s chi-square test for comparison of proportions. Statistical significance was set at *p* < 0.05.

## Results

### Characteristics of the study population

One thousand six hundred nine (1609) children met the inclusion criteria and were enrolled; 318, 318, 341, 315 and 317 were enrolled in Bamenda, Limbe, Maroua, Ngaoundere and Yaounde, respectively (Table 1). Among them were 779 (48.4%) females and 830 (51.6%) males. The ages of the participants ranged between 0 and 180 months (mean ± SD = 63.65 ± 56.69).

**Table 1:** Distribution of the study population with respect to age, gender and study site

Epidemiological strata	Study sites			Age (months)			Total
				<60	60–119	120+	
HWP	Bamenda	Gender	F	70 (40.5)	29 (16.8)	74 (42.8)	173 (54.4)
			M	58 (40.0)	38 (26.2)	49 (33.8)	145 (45.6)
		Total		128 (40.3)	67 (21.1)	123 (38.7)	318
C	Limbe	Gender	F	75 (50.3)	39 (26.2)	35 (23.5)	149 (46.9)
			M	103 (61.0)	39 (23.1)	27 (15.9)	169 (53.1)
		Total		178 (56.0)	78 (24.5)	62 (19.5)	318
SCEF	Yaounde	Gender	F	61 (40.7)	32 (21.3)	57 (38.0)	150 (44.0)
			M	100 (52.4)	46 (24.1)	45 (23.6)	191 (56.0)
		Total		161 (47.2)	78 (22.9)	102 (29.9)	341
SS	Maroua	Gender	F	122 (74.4)	27 (16.5)	15 (9.1)	164 (52.1)
			M	111 (73.5)	27 (17.9)	13 (8.6)	151 (47.9)
		Total		233 (74.0)	54 (17.1)	28 (8.9)	315
HIP	Ngaoundere	Gender	F	94 (65.7)	32 (22.4)	17 (11.9)	143 (45.1)
			M	116 (66.7)	34 (19.5)	24 (13.8)	174 (54.9)
		Total		210 (66.3)	66 (20.8)	41 (12.9)	317
Total		Gender	F	422 (54.2)	159 (20.4)	198 (25.4)	779 (48.4)
			M	488 (58.8)	184 (22.2)	158 (19.0)	830 (51.6)
		Total		910 (56.6)	343 (21.3)	356 (22.1)	1609

Data are presented as number (%)

*HWP* High western plateau strata, *C* Coastal strata, *SCEF* South Cameroonian Equatorial strata, *SS* Sudano-sahelian strata, *HIP* High inland plateau strata, *F* female, *M* male

### Prevalence of malaria in the study population

Among the 1609 participants, 242 and 283 were positive for malaria parasites by Giemsa microscopy (GM) and PCR, respectively. A marginal difference was observed in the detection rate between GM and PCR (*p* = 0.05). However, GM demonstrated a high sensitivity (85.5%) and specificity (100%) and, overall, a perfectly correlated agreement with PCR (97.5%) in the current study (Table 2). The PCR-adjusted prevalence was 17.6% (95% CI 15.8–19.5). The prevalence of malaria was highest in Limbe 29.9% (95/318; 95% CI 24.9–35.2) followed by Yaounde 21.9% (75/341; 95% CI 17.7–26.7), Bamenda 16.7% (53/318; 95% CI 12.7–21.2) and Ngaoundere 10.7% (34/317; 95% CI 7.5–14.7) and lowest in Maroua 8.3% (26/315; 95% CI 5.5–11.9). There was a significant association between the malaria prevalence and study site (*p* < 0.001).

**Table 2:** The performance of Giemsa microscopy (GM) in comparison to PCR in the detection of malaria parasites in the study population

		PCR		
		Positive	Negative	Total
Giemsa microscopy	Positive	242	0	242
	Negative	41	1326	1367
	Total	283	1326	1609
Sensitivity, % (CI)	85.5 (80.9–89.4)			
Specificity, %	100			
Positive predictive value (PPV), %	100			
Negative predictive value (NPV), % (CI)	97.0 (96.0–97.8)			
False positive rate (FPR), % (CI)	0.0 (0.0–2.3)			
False negative rate (FNR), % (CI)	14.5 (2.2–40.1)			
Agreement between tests, % (CI)	97.5 (96.6–98.2)			

Sensitivity = [true positive/(true positive + false negative) × 100]; specificity = [true negative/(true negative + false positive) × 100; PPV = [true positive/(true positive + false positive) × 100]; NPV = [true negative/(true negative + false negative) × 100]; Agreement = [true positive + true negative/N × 100]; FPR = 1 − specificity; FNR = 1 − sensitivity

Overall, there was no significant association between the prevalence of malaria and gender (*p* = 0.239, Table 3).

**Table 3:** Distribution of malaria in the study population stratified according to age, gender and study site

Study site	Gender						Age category (months)							
	Female		Male		*χ* ^2^	*p* value	<60		60–119		120+		*χ* ^2^	*p* value
	*n*	Pos (%)	*n*	Pos (%)			*n*	Pos (%)	*n*	Pos (%)	*n*	Pos (%)		
Bamenda	173	34 (19.7)	145	19 (13.1)	2.436	0.119	128	16 (12.5)	67	16 (23.9)	123	21 (17.1)	4.125	0.127
Limbe	149	48 (32.2)	169	47 (27.8)	0.733	0.392	178	46 (25.8)	78	34 (43.6)	62	15 (24.2)	9.340	0.009
Yaounde	150	32 (21.3)	191	43 (22.5)	0.068	0.794	161	32 (19.9)	78	23 (29.5)	102	20 (19.6)	3.312	0.191
Maroua	164	15 (9.2)	151	11 (7.3)	0.360	0.549	232	19 (8.2)	54	5 (9.3)	28	2 (7.1)	0.121	0.941
Ngaoundere	143	17 (11.9)	174	17 (9.8)	0.368	0.544	210	22 (10.5)	66	7 (10.6)	41	5 (12.2)	0.167	0.948
Total	779	146 (18.7)	830	137 (16.5)	1.386	0.239	910	135 (14.8)	343	85 (24.8)	356	63 (17.7)	17.005	<0.001

Generally, the prevalence of malaria was highest in participants between 60 and 119 months and lowest in participants below 60 months (Table 3). A significant association was observed between the prevalence of malaria and age (*p* < 0.001).

All (100%) the cases of malaria in this study were caused by *P. falciparum* as determined by the multiplex PCR and GM. Neither *P. vivax*, *P. malariae*, *P. oval*e nor *P. knowlesi* was identified. Furthermore, no mix infection with other *Plasmodium* species was observed.

## Discussion

In the current study, the overall prevalence of malaria was 17.6% as determined by PCR. This prevalence is however lower than the national prevalence of 29% reported in 2012 [22]. This difference could be attributed to the relentless efforts of Cameroon’s government to control the disease through the mass distribution of insecticide-treated bed nets to all the households in the country and also to the intense sensitization of the population through media [29, 30]. The prevalence of malaria was highest in Limbe (in the C strata) and lowest in Maroua (in the SS strata). The decreasing trend observed in the prevalence of malaria from the South towards the North of the country could be attributed to the geographical setting; transmission of malaria has been described as hyperendemic in Limbe [31], holoendemic in Yaounde [30, 32], mesoendemic in Bamenda and Ngaoundere and hypoendemic in Maroua. The prevalence of malaria did not differ between males and females, which is consistent with other studies [4, 22, 33–36]. On the other hand, the prevalence of malaria was significantly higher in children between 60 and 119 months of age and this is consistent with some other studies [33, 37, 38]. The higher prevalence of malaria in children between 60 and 119 months could be attributed to their playful attitude as they grow up which exposes them to infective bites of the *Anopheles* mosquitoes. The observation of a significant association between the prevalence of malaria and age in the current study is however contrary to some studies that found no association of malaria with age [26–34].

In the current study, *P. falciparum* was the only parasite species identified. This is reminiscent of the WHO report that all (100%) of malaria cases in Cameroon are caused by *P. falciparum* [39]. It is also consistent with studies which show that *P. falciparum* was associated with all the cases of clinical malaria in the country [15–17]. The finding is however contradictory to studies that have reported prevalences of *P. vivax* ranging from 4% [14] to 14.9% [13]. The difference between these studies and ours could be attributed to differences in the study design; our study targeted children presenting with symptoms of malaria meanwhile theirs targeted both symptomatic or asymptomatic adults and children. In addition, the differences in the respective study locations could account for the differences in the results obtained. Furthermore, no mixed infection was detected in the current study, which is contrary to other studies that have reported low prevalence of mixed infection between *P. falciparum* and *P. malariae* or *P. ovale* [15–17]. The difference between these studies and ours could also be attributed to the study design; Giemsa microscopy was the tool used to identify the different species of Plasmodia which is subject to errors especially when not done by experts [40, 41]; meanwhile, in our study, PCR was used. The high sensitivity of PCR is further demonstrated in this study as the detection rate was significantly higher with PCR compared to that with microscopy. This discrepancy could stem from differences in the detection limits of microscopy and PCR; microscopy has a detection limit of 50–100 parasites/μl; meanwhile, PCR has a detection limit as low as 1–5 parasites/μl [42, 43]. PCR has the added advantage of accurately detecting the different plasmodia species [42, 44, 45]. However, GM in the current study demonstrated a perfectly correlated agreement with PCR (97.5%). In the current study, *P. falciparum* was correctly identified as the only *Plasmodium* species by GM and this confirms the high specificity of GM in the identification of the different Plasmodia. One limitation of using PCR in the diagnosis of malaria is that the parasite DNA can remain in the blood stream long after the infection has been cleared and therefore differentiating an active infection from a recently cleared infection poses a challenge [46]. As a consequence, microscopy still remains the gold standard [41, 47, 48]. In the current study, no infection with *P. knowlesi* was observed. Being one of the first studies to target *P. knowlesi* in the country serves as a confirmation of its absence.

In this study, data was collected only during the rainy season during which transmission is usually at its peak. This may have affected the distribution of the different Plasmodia. Studies designed to collect data in both the rainy and the dry season will be paramount to providing a clearer picture of the distribution of the Plasmodia with season. More so, in the current study, participants were recruited only from health care facilities in urban settings and the data generated may therefore not be generalisable to children living in rural areas. Studies will also be needed in rural areas to give a clearer picture.

## Conclusions

A malaria prevalence of 17.6% was observed in the target population. The prevalence was significantly higher in children between 60 and 119 months. On the contrary, gender had no influence on malaria prevalence. A significant association was also observed between the prevalence of malaria and the geographical setting, being highest in Limbe (in the C strata) and lowest in Maroua (in the SS strata). *P. falciparum* was the only species associated with clinical malaria in the target population, and no mixed infection with other *Plasmodium* species was identified, which is contrary to earlier studies.

## Abbreviations

C: Coastal strata
FNR: False negative rate
FPR: False positive rate
GM: Giemsa microscopy
HIP: High inland plateau strata
HWP: High western plateau strata
NPV: Negative predictive value
P: Plasmodium
PCR: Polymerase chain reaction
PPV: Positive predictive value
SCEF: South Cameroonian Equatorial strata
SS: Sudano-sahelian strata
